# Complete Genome Sequence of *Bacillus paralicheniformis* MDJK30, a Plant Growth-Promoting Rhizobacterium with Antifungal Activity

**DOI:** 10.1128/genomeA.00577-17

**Published:** 2017-06-22

**Authors:** Yun Wang, Hu Liu, Kai Liu, Chengqiang Wang, Hailin Ma, Yuhuan Li, Qihui Hou, Fangchun Liu, Tongrui Zhang, Haide Wang, Beibei Wang, Jinjin Ma, Ruofei Ge, Baochao Xu, Gan Yao, Wenfeng Xu, Lingchao Fan, Yanqin Ding, Binghai Du

**Affiliations:** a College of Life Sciences/Shandong Key Laboratory of Agricultural Microbiology/National Engineering Laboratory for Efficient Utilization of Soil and Fertilizer Resources, Shandong Agricultural University, Tai’an, China; b Shandong Academy of Forestry, Jinan, China; c College of Resources and Environment, Shandong Agricultural University, Tai’an, China; d State Key Laboratory of Nutrition Resources Integrated Utilization, Linshu, China

## Abstract

*Bacillus paralicheniformis* MDJK30 was isolated from the rhizosphere of a peony. It could control the pathogen of peony root rot. Here, we report the complete genome sequence of *B. paralicheniformis* MDJK30. Eleven secondary metabolism gene clusters were predicted.

## GENOME ANNOUNCEMENT

The *Bacillus* genus comprises typical species of plant growth-promoting rhizobacteria (PGPRs) that are able to suppress some plant pathogens by producing antagonistic substances. For instance, *B. subtilis* RB14-CS can inhibit the plant pathogen *Rhizoctonia solani* by exerting iturin A (1). The difficidin (2), bacilysin (3), and surfactin (4) produced by *B. amyloliquefaciens* are considered to be beneficial compounds against plant pathogens. *B. paralicheniformis* is a Gram-positive species of the *Bacillus* genus. Rubén Palacio-Rodríguez et al. reported that *B. paralicheniformis* LBEndo1 can promote the growth of *Arabidopsis thaliana* (5). *B. paralicheniformis* MDJK30 was isolated from the rhizosphere of peony in Shandong, China. It has the ability to suppress *Fusarium solani*, which can cause root rot in peonies.

The whole genome of *B. paralicheniformis* MDJK30 was sequenced using the Illumina MiSeq and PacBio RS II platforms. We obtained 5,106,074 high-quality reads through the Illumina MiSeq platform and 996,186 reads through the PacBio RS II platform. The coverage of the sequence reached 263×. All reads produced with the Illumina MiSeq were *de novo* assembled using Newbler version 2.8 (20110517_1502) (6), and those produced with the PacBio RS II were assembled with FALCON-integrate version 0.3.0. The annotation of the complete genome sequence was conducted using the NCBI Prokaryotic Genome Annotation Pipeline (http://www.ncbi.nlm.nih.gov/genome/annotation_prok). The clustered regularly interspaced short palindromic repeats (CRISPRs) were predicted with the CRISPR recognition tool (CRT) (7). The PHAge search tool (PHAST) was utilized to find the prophages (8). The analysis of carbohydrate-active enzymes was carried out using the Carbohydrate-Active enZYmes database (9). Genomic islands were predicted using IslandViewer (10). The secondary metabolisms were predicted with antiSMASH (11) version 3.0.5 (http://antismash.secondarymetabolites.org).

The circular chromosome of *B. paralicheniformis* MDJK30 consists of 4,352,468 bp, and the G+C content is 45.94%. A total of 4,363 genes were annotated, including 4,134 coding genes, 110 RNA genes, and 119 pseudogenes. The RNA genes were composed of 24 rRNAs, 81 tRNAs, and 5 ncRNAs. Two CRISPRs and one prophage were discovered. The analysis of the genome showed that 166 genes were related to code carbohydrate-active enzymes, 71 genes coding auxiliary activities, 39 genes related to carbohydrate-binding modules, 9 genes coding carbohydrate esterases, 34 genes relevant to glycoside hydrolases, 5 genes coding glycosyl transferases, and 25 genes coding polysaccharide lyases. A total of 22 genomic islands were predicted, and 220 annotated genes were found in them. Eleven gene clusters related to secondary metabolism were predicted. The gene cluster (BLMD_02100-BLMD_02320) was 100% similar to that of lichenysin biosynthesis genes. The gene cluster (BLMD_12845-BLMD_13070) was 100% similar to that of bacitracin biosynthesis genes. These two gene clusters both belonged to nonribosomal peptide synthetases. The other gene clusters might be related to the production of new antimicrobial compounds. The complete genome of *B. paralicheniformis* MDJK30 will be helpful for studying the mechanisms of plant growth promotion and biocontrol at the molecular level.

### Accession number(s).

The genomic sequence of *B. paralicheniformis* MDJK30 has been deposited at GenBank under the accession number CP020352.
